# Investigation of intraocular pressure of the anterior chamber and vitreous cavity of porcine eyes via a novel method

**DOI:** 10.1038/s41598-020-77633-7

**Published:** 2020-11-25

**Authors:** Koji Nagae, Hiromasa Sawamura, Makoto Aihara

**Affiliations:** Department of Ophthalmology, Graduate School of Medicine, The University of Tokyo, 7-3-1, Hongo, Bunkyo-ku, Tokyo, 113-8654 Japan

**Keywords:** Biophysics, Anatomy, Medical research

## Abstract

To evaluate a new method of measuring the intraocular pressure (IOP) in the vitreous cavity. IOPs in the anterior chamber and vitreous cavities of 24 porcine eyes (12 eyes with lenses and 12 eyes without lenses) were measured directly, continuously, and simultaneously. We used a needle as a part of the pressure sensor to measure the anterior chamber IOP and a disk-shaped sensor to measure the vitreous cavity IOP. A significant group-by-condition interaction on the vitreous cavity IOP between the two groups (phakia and aphakia) and four conditions of anterior chamber IOP were observed (F[3,258] = 5.8564, p < 0.001). A positive correlation was observed between the vitreous cavity IOP and anterior chamber IOP in both the phakia group (R = 0.96, p < 0.001) and the aphakia group (R = 0.97, p < 0.001). No significant correlation was observed between the ΔIOPv-a (vitreous cavity IOP − anterior chamber IOP) and anterior chamber IOP in either group (phakia group: R =  − 0.18, p = 0.034; aphakia group: R =  − 0.029, p = 0.73). The vitreous cavity IOP measured with the new sensor was well-correlated with the anterior chamber IOP in the physiological range tested.

## Introduction

Intraocular pressure (IOP) is derived from aqueous dynamics and physiologically sustains the eyeball. The eyeball, except for the optic disk, is completely covered by a collagen matrix. IOP is necessary for biological activities associated with visual function, as it helps to maintain the eye’s structure as a whole. For example, retinal fold or choroidal detachment may occur if IOP is reduced.

Direct measurement of IOP is one of several indices used to evaluate drug efficacy^1^ and pathophysiology^2^ in animal models of glaucoma. By contrast, indirect measurement of IOP (outside the corneal surface) is conducted clinically using various methods, and its value has been recognized as being representative of eye pressure. It has been reported that IOP is often underestimated when measured indirectly with an applanation tonometer, but is well-correlated with the IOP measured directly in the anterior chamber of rabbit^3^ and porcine^4^ eyes. The intraocular space is separated by the lens of the eye, and the anterior part is irrigated by the aqueous humor. The intraocular space contains the anterior chamber and the posterior chamber, and the posterior part (the vitreous cavity) is occupied by the vitreous body and its degraded liquid. As such, two factors, the presence of the lens and the different components of the anterior part and posterior part, may affect the IOP distribution, resulting in a difference in the IOP between the anterior chamber and the vitreous cavity. If differences in IOP exist between the anterior chamber and vitreous cavity, it is crucial to determine the degree of this difference, considering that indirectly measured IOPs are used as a therapeutic index, especially in glaucoma.

Previous studies have investigated the differences in IOP using direct measurement of the IOP in both the anterior chamber and the vitreous cavity of porcine eyes^5,6^. One group reported the differences in the IOP between the anterior chamber and vitreous cavity; however, the pressure in the vitreous cavity could not be completely evaluated because of the viscosity of the vitreous gel^5^. Other researchers have reported that, under a certain degree of pressure (< 50 mmHg), the IOP does not differ significantly between the anterior chamber and vitreous cavity without vitreous gels^6^. Therefore, in this case, the lens had little effect on the differences in IOP. However, given that the vitreous gel was removed in the above-mentioned study, the effect of the lens when the vitreous gel is present in the vitreous cavity remains unclear.

A newly developed waterproof device without a tubular structure was considered able to measure the pressure in the vitreous gel without clogging and was used to monitor the IOP in the vitreous cavity to address the viscosity of the vitreous body. A method to measure the pressure applied to a gel via a strain change has been reported previously^7^. To clarify the accuracy of the device, we modified the method partially and performed a control experiment to evaluate how accurately the device measures the pressure in a gel by enclosing it in an artificial gel and measuring the water pressure. The objective was to measure the IOPs of the anterior chamber and vitreous cavity without removal of the vitreous gel and to evaluate the usefulness of the device from the correlation of the IOPs using direct and simultaneous measurements of the IOP in both areas of the eye.

## Methods

### Animal eyes

Twenty-four freshly enucleated porcine eyes were obtained from Tokyosibaurazouki Corporation (Tokyo, Japan). The following experiments were conducted at room temperature within 12 h of enucleation. IRB/ethics committee approval was not required as eyes that would be discarded at the meat market were used.

### Measurement of the water pressure using a disc-shaped pressure sensor enclosed in an artificial gel

To verify the accuracy of the pressure measurement in the vitreous gel, we measured the pressure in the artificial gel with a disk-shaped pressure sensor (PDA-PB; Tokyo Measuring Instruments Lab, Tokyo, Japan). We prepared a gel with 98% moisture content using gelatin, salt, and water. The sensor was enclosed in the gel with a size of 2 cm on each side, and the gel was placed in a thin polyethylene plastic bag. The plastic bag was submerged into water to prevent water from flowing in. After installing the sensor at water depths of 10, 15, and 20 cm (the true water pressure was 7.35, 11.0, and 14.7 mmHg) and after waiting for the values to stabilize, we measured the pressure inside the gel. It was thought that the pressure inside the gel would be close to the water pressure outside the plastic bag, and the accuracy of the sensor could be confirmed by comparing the true water pressure with the measured value for reference. This experiment was performed three times.

### IOP measurement in the anterior chamber and vitreous cavity

We used a custom-designed device with a 27-gauge needle attached to the tip of a pressure gauge (KDM30; Krone Corp., Tokyo, Japan) to measure the water pressure at the tip of the needle and send data to a computer wirelessly. The tip of the needle was secured in the anterior chamber of the eyes, and continuous measurements were recorded as the anterior IOP. To record the IOP of the vitreous cavity, we made a 10-mm incision in the sclera along the equator and inserted a disk-shaped pressure sensor in the vitreous gel. The incision was then sutured to prevent leakage of the vitreous gel, so that only the cable connecting the sensor was passed through the incision. The sensor was connected to the computer via a transducer (DC-004P; Tokyo Measuring Instruments Lab), which converts a change in electrical resistance caused by deformation of the pressure sensor into a change of pressure value, and continuous measurements were recorded. This testing configuration allowed for IOP measurement, even in the vitreous gel, as the vitreous IOP. All gauges and sensors were used after confirming that they could accurately measure the pressure of 2 cm (1.5 mmHg) of water by zeroing under atmospheric pressure. The measured values of the gauges and sensors indicate the change in the absolute pressure since the zero point correction. Therefore, if the atmospheric pressure changes during the experiment, the change will be reflected in the measured values, but the difference between the two IOP values does not contain the atmospheric pressure change.

It is considered that the pressure is not constant in the anterior chamber or in the vitreous cavity, and the pressure value varies with the measurement position due to gravity, the flow of aqueous humor, and deformation of the vitreous body. In this experiment, the anterior IOP and the vitreous IOP do not indicate the representative values of each chamber, but show the measurement values from each chamber.

### Simultaneous and continuous measurement of the anterior IOP and vitreous IOP

**Figure 1:**
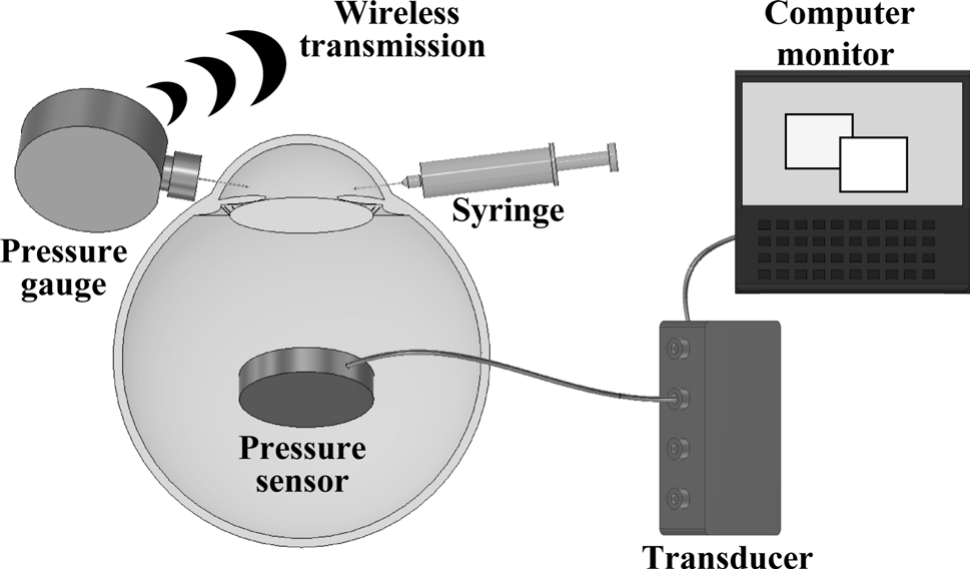
Schematic illustration of the simultaneous measurement of the intraocular pressures (IOPs) in the anterior chamber and vitreous cavity. Pressure measurements in the anterior chamber and vitreous cavity were performed in an upright position simultaneously and continuously. The two IOPs were shown on the computer monitor and recorded during the experiment. The pressure gauge in the anterior chamber was connected wirelessly, and the pressure sensor in the vitreous cavity was connected to the computer via the transducer.

We placed the porcine eyes in an upright position. After positioning the pressure sensor in the vitreous cavity, two 27-gauge needles were inserted and placed in the anterior chamber. One was connected to an injection syringe to increase the anterior IOP to around 50 mmHg. One needle was held for a few seconds by hand before measuring, and another needle was attached to the pressure gauge (Fig. 1). The injection syringe and pressure gauge were fixed so that they could not move or be touched during measuring. The anterior IOP and the vitreous IOP were recorded simultaneously and continuously after injecting saline into the anterior chamber. To measure the IOP of aphakic eyes, phacoemulsification and aspiration were conducted using the Whitestar system (Whitestar Signature Pro System; Johnson & Johnson Surgical Vision Inc., Irvine, CA, USA) to remove the lens without damaging the posterior capsule. All incisions made to insert the devices were sutured. Subsequently, for phakic eyes, the pressure sensors were inserted similarly. The experiments were conducted while carefully observing that there was no leakage from the incisions.

### Measurement of the ΔIOPv-a in phakic and aphakic eyes

A total of 24 porcine eyes, 12 with lenses (phakia group) and 12 without lenses (aphakia group) were used to evaluate the differences in IOP. Saline was injected into the anterior chamber until IOP increased to about 50 mmHg; thereafter, the anterior IOP gradually decreased to 10 mmHg. When the anterior IOP reached each targeted pressure value (40, 30, 20, and 10 mmHg), the vitreous IOP was subtracted from the anterior IOP to calculate the ΔIOPv-a. Experiments were repeated twice by injecting again a few minutes after the end of the earlier experiment.

### Statistics

All values are indicated as mean ± standard deviation. Statistical analysis was conducted using JMP software (version 15; SAS Institute Inc., Cary, NC, USA). Two-factorial repeated-measurement analysis of variance (ANOVA) was conducted with the vitreous IOP as the dependent variable, with two groups (phakia and aphakia) and four conditions of anterior IOP (40, 30, 20, and 10 mmHg) as the two main factors. We also conducted a linear regression analysis to determine the correlation between the anterior IOP and vitreous IOP and the stability of the ΔIOPv-a in the phakia and aphakia groups separately. A p-value of < 0.025 was considered statistically significant after Bonferroni correction.

## Results

### The measurement of the water pressure in the gel using a disc-shaped pressure sensor

The average water pressure values measured by a disc-shaped pressure sensor dipped in the gel are summarized in Table 1. In each true water pressure of 7.35, 11.0, and 14.7 mmHg, the disc-shaped sensor indicated 6.68, 10.5, and 14.2 mmHg. The difference between the values of measured water pressure value and true water pressure was 0.5–0.67 mmHg. The disc-shaped pressure sensor demonstrated high accuracy and less variation in the measurement of pressure.

**Table 1:** Verification of the pressure measurement in the gel with a disc-shaped pressure sensor (n = 3).

True water pressure (mmHg)	Mean measured values of a disc-shaped pressure sensor (mmHg)
7.35	6.68 (6.68, 6.68, 6.68)
11.0	10.5 (9.98, 10.8, 10.8)
14.7	14.2 (14.2, 14.2, 14.2)

### The difference between vitreous IOP and anterior IOP in the phakia and aphakia groups

The average vitreous IOP values in the phakia and aphakia groups are listed in Table 2. A repeated-measures ANOVA revealed significant differences in the vitreous IOPs between the anterior IOP conditions (F[3,258] = 13,610.70, p < 0.001), and a significant group-by-condition interaction (F[3,258] = 5.8564, p < 0.001). No significant difference was observed between the phakia and aphakia groups (F[1,22] = 3.3645, p = 0.080).

**Table 2:** Vitreous cavity IOP in the phakia group and aphakia group when anterior chamber IOP reached each target pressure.

Anterior IOP (mmHg)	Vitreous IOP (mmHg) (mean IOP ± SD)	
	Phakia (n = 12)	Aphakia (n = 12)
40	44.2 ± 3.61	42.7 ± 3.17
30	34.9 ± 2.97	33.0 ± 2.85
20	25.1 ± 2.69	23.0 ± 2.61
10	15.7 ± 2.11	12.9 ± 2.22

**Figure 2:**
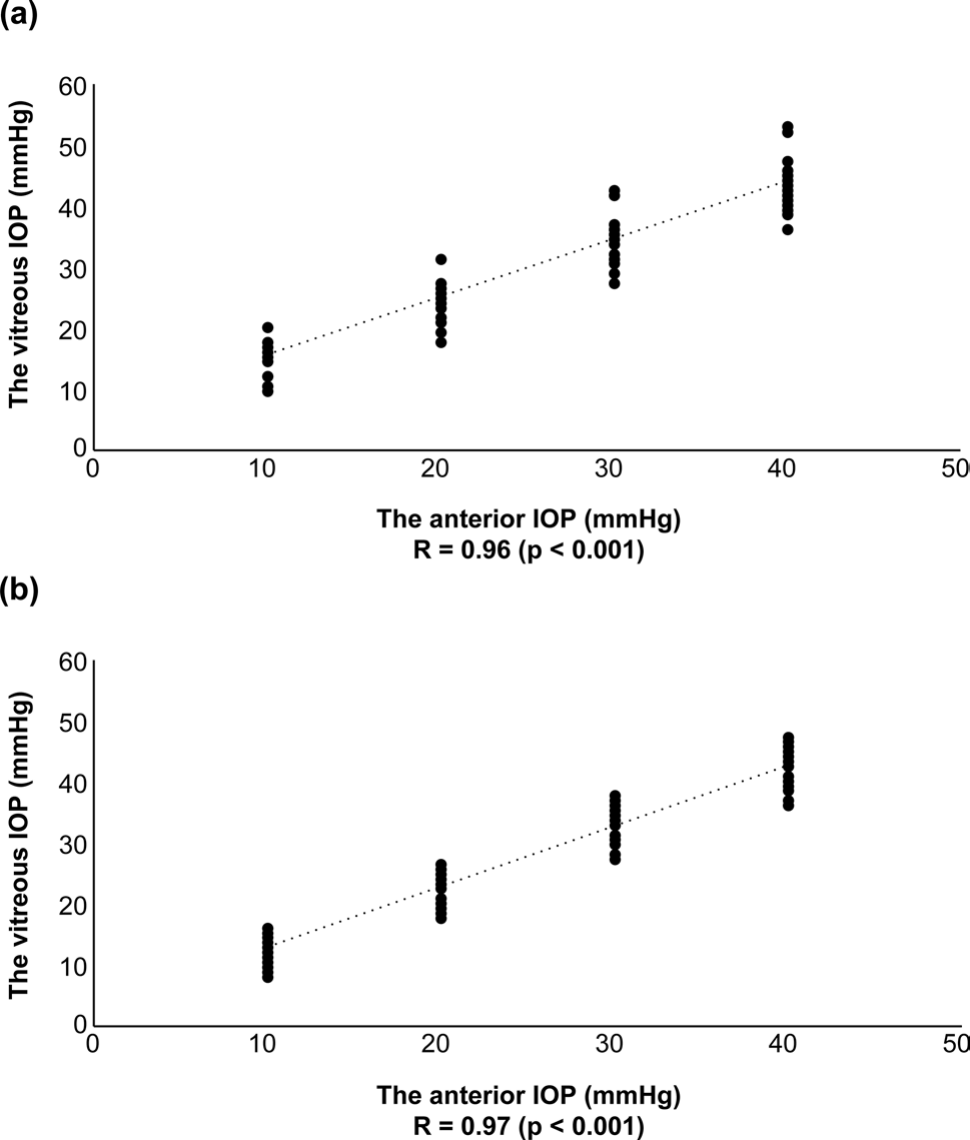
Scatterplots of the vitreous cavity IOP under different anterior chamber IOP (abscissa) in the phakia group (n = 12) (**a**) and aphakia group (n = 12) (**b**). The vitreous IOP was well-correlated with the anterior IOP in both groups.

**Figure 3:**
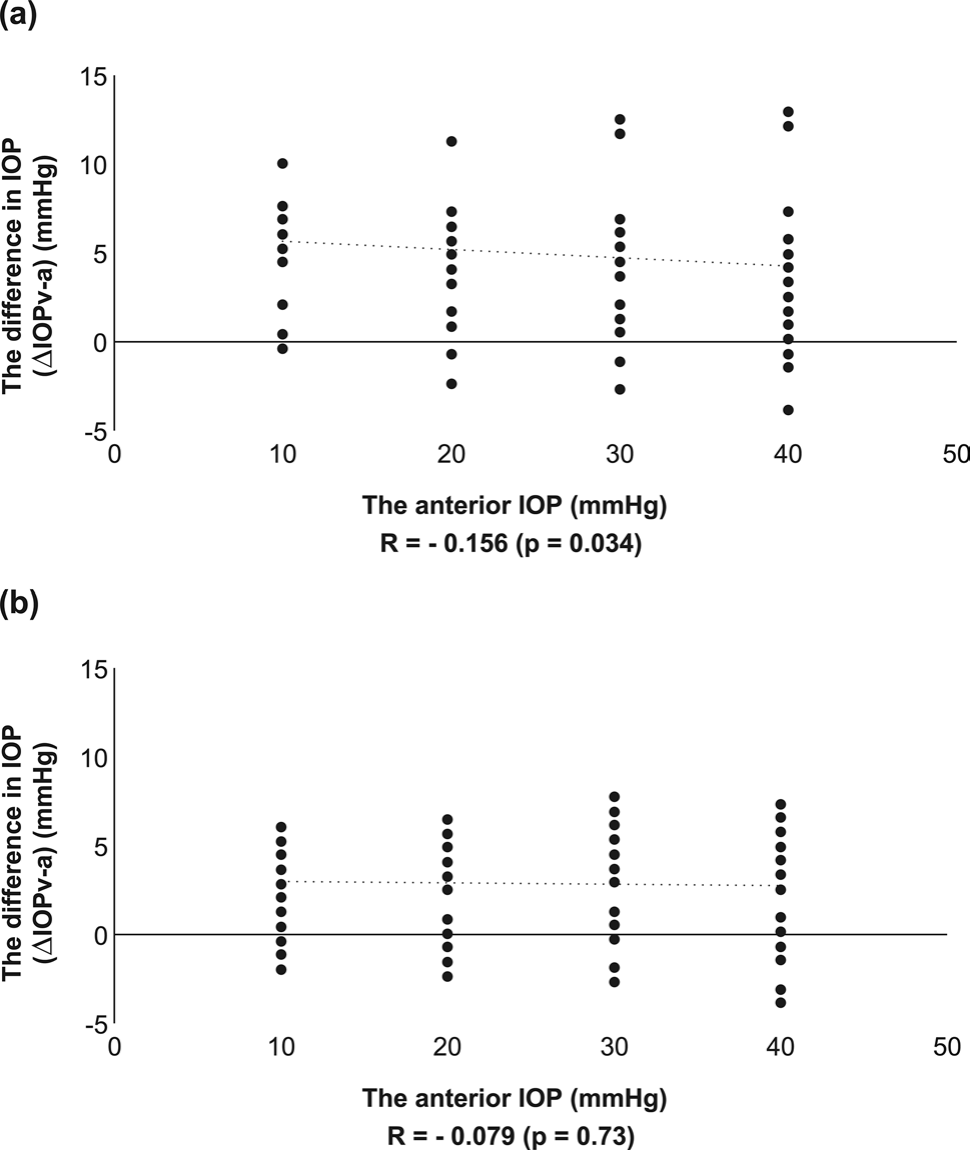
Scatterplots of the difference in IOPs between the anterior chamber and vitreous cavity (ΔIOPv-a, ordinate) under different anterior chamber IOP (abscissa) in the phakia group (n = 12) (**a**) and aphakia group (n = 12) (**b**). The ΔIOPv-a was not correlated with the anterior IOP in either group.

To investigate the correlation between anterior IOP and vitreous IOP and the stability of the ΔIOPv-a in the phakia and aphakia groups, linear regression analyses were conducted between the vitreous IOP and anterior IOP and between the ΔIOPv-a and anterior IOP for the phakia and aphakia groups separately. A positive correlation between the vitreous IOP and anterior IOP was observed in the phakia group (Fig. 2a; correlation coefficient [R] = 0.96, p < 0.001) and the aphakia group (Fig. 2b; R = 0.97, p < 0.001), showing that the vitreous IOP was well-correlated with the anterior IOP in both groups. Interestingly, ΔIOPv-a was positive in four different anterior IOP and stable, around 4–5 mmHg in the phakia group, and 3 mmHg in the aphakia group. No correlation was found between the ΔIOPv-a and anterior IOP in the phakia group (Fig. 3a; R =  − 0.18, p = 0.034) or the aphakia group (Fig. 3b; R =  − 0.029, p = 0.73), indicating that the ΔIOPv-a was not correlated with the anterior IOP in either group in the examined range.

## Discussion

The present study shows that the new device without a tubular structure, which was considered to prevent clogging, could measure the vitreous IOP in the vitreous body directly and that vitreous IOP was well-correlated with the anterior IOP in the physiological range studied. However, the ΔIOPv-a did not significantly correlate with the anterior IOP in either the phakia or the aphakia group in the range examined.

As a result of the verification experiment, it was confirmed that the new device can be used for measurement in a gel. Thus, we believe that the vitreous IOP in our research was considered close to the true vitreous IOP.

In the present experiment, almost all the ΔIOPv-a values were positive in both the phakia and aphakia groups. The similar ΔIOPv-a values indicated a gravity effect. Since the eyeballs were placed in the upright position, there was a height difference between the two tips of the sensors, as shown in Fig. 1. However, gravity alone can only explain a water pressure of up to about 24 mm (1.82 mmHg) because the height difference was considered to be the maximum if it was the axial length of the eyes^8^. The ΔIOPv-a in both groups was greater than 1.82 mmHg, and other factors were considered besides gravity. One possible explanation is that a non-physiological condition of the vitreous cavity was created by inserting the sensor and the enlarged volume of the vitreous cavity increased the vitreous IOP, but the anterior IOP could not increase as much as the vitreous IOP. The vitreous gel of aphakic eyes could deform toward the anterior chamber due to the loose tension of the capsule, and the capsule cannot suppress gel movement as well as the lens does. The vitreous IOP of aphakic eyes may not increase as much as that of phakic eyes even with the same sensor inserted. It was considered that the lens functioned as a partition between the anterior chamber and vitreous cavity and the partition effect on the ΔIOPv-a was not constant and changed along with changes in the anterior IOP, resulting in a significant group-by-condition interaction, although no significant differences were observed between phakia and aphakia groups.

Previously, the ΔIOPv-a of porcine eyes has been reported^5^; specifically, the vitreous IOP was 55 mmHg (range, 16–68) when the anterior IOP was 135 mmHg (range, 122–145). The authors concluded that the large ΔIOPv-a (80 mmHg) might be due to the vitreous gel clogging the tube of the pressure sensor. In the present experiment, the pressure sensor did not have a tubular structure, which allowed measurement of the vitreous IOP close to the anterior IOP, even in the presence of vitreous gel.

Another study using porcine eyes with the vitreous gel removed showed no difference in IOP between the anterior chamber and vitreous cavity when the vitreous IOP was less than 50 mmHg^6^. These results correlate with our results and suggest that the presence of vitreous gel and/or the different methods did not affect the ΔIOPv-a values significantly. Our report is noteworthy as it is the first to evaluate the differences in IOP between the anterior chamber and vitreous cavity in porcine eyes with the vitreous gel preserved.

IOP is affected by aqueous humor dynamics, especially in places with narrow channels^9^. Bernoulli’s theorem is shown as

$$ {\text{P }} + { }\frac{1}{2}{\rho V}^{2} { } + {\uprho\text{gh}} = {\text{C,}} $$

where P (Pa, 1 mmHg ≓ 133 Pa) is the pressure at one position, ρ (1000 kg/m^3^) is the fluid density, V (m/s) is the fluid velocity, g (9.8 m/s^2^) is the acceleration of gravity, h (m) is its height, and C (Pa) is constant. In the present study, P (the anterior IOP) ranged from 10 to 40 mmHg or 1333 to 5333 Pa. The third term ($\uprho {\text{gh}}$) represents the potential energy, which was considered to be about 9.8 Pa per 1 mm height change, much lower than P. The second term ($\frac{1}{2}{\rho V}^{2}$), which represents the kinetic energy, was 0.05, 5.00, and 500 Pa if V was 0.01, 0.10, and 1.00 m/s, respectively. Although the velocity near the needle was not measured in the present study, the effect of the flow on the pressure measurement in the anterior chamber was considered to be negligible because the velocity 0.10 m/s (100 mm/s) meaning the second term 5.00 Pa and much lower than P was not realistic in the chamber after injection. The result that the ΔIOPv-a in the aphakia group was almost constant also suggested that the influence of the flow was negligible because the velocity was considered to be slowing as the anterior IOP decreased. From the above, it was considered that the influences of the flow of saline and the height of the needle were negligible in the present study.

The importance of the measurement of the ΔIOPv-a cannot be overemphasized in considering the progression of glaucoma. In clinical practice, IOP values measured with applanation tonometers are used as therapeutic effect markers. However, our results indicate that a difference in IOP between the anterior chamber and the vitreous cavity could occur to be greater than the gravity effect. Therefore, the real vitreous IOP, which affects the extent of damage to the optic disk, cannot always be reflected in the measurements of the anterior IOP. Furthermore, the real vitreous IOP may be underestimated if medical or pathological conditions occur in the vitreous chamber or lens. Additionally, the relationship between IOP and the progression of myopia with axial elongation has been reported in animal experiments using young guinea pigs^10^, suggesting that IOP has an effect on myopia progression; therefore, evaluating the real vitreous IOP may be as important in myopia as it is in glaucoma.

The present study has several limitations. First, only the ΔIOPv-a in the supine position was reflected because the eyeballs were facing upward during the experiments. How the ΔIOPv-a changes with position (e.g., the sitting position or with eye movement) is unclear. Second, the IOP in the posterior chamber between the iris and the lens should be measured; however, sensor placement in this area was difficult. Third, the possibility of the pressure difference decreasing due to physical changes in tissues, such as the sclera or vitreous gel, was not considered.

In conclusion, the present study shows that the vitreous IOP can be directly measured with a device without a tubular structure and that this measurement correlates well with the anterior IOP. We have also demonstrated that the ΔIOPv-a can be partially created by experimental conditions affected by the gravity, but ΔIOPv-a may be present in the physiological condition. As sensors become smaller and wireless capabilities become available, it may be possible to place the sensors in the anterior chamber or vitreous cavity of animals for a longer duration, allowing for more precise measurements and new physiological findings.

## Data Availability

The datasets generated during and/or analyzed during the current study are available from the corresponding author on reasonable request.
